# Health-related costs in a sample of premenopausal non-diabetic overweight or obese females in Antwerp region: a cost-of-illness analysis

**DOI:** 10.1186/s13690-018-0285-1

**Published:** 2018-07-30

**Authors:** W. Hens, D. Vissers, L. Annemans, J. Gielen, L. Van Gaal, J. Taeymans, N. Verhaeghe

**Affiliations:** 1 Faculty of Medicine and Health Sciences, University of Antwerp, Antwerp, Belgium; 2 Faculty of Medicine & Health Science, Ghent University, Ghent, Belgium; 3 Department of Radiology, Antwerp University Hospital, Antwerp, Belgium; 4 Department of Endocrinology, Antwerp University Hospital, Diabetology and Metabolism, Antwerp, Belgium; 5 Bern University of Applied Sciences-Health, Bern, Switzerland; 6 Faculty of Sport and Rehabilitation Sciences, Vrije Universiteit Brussel, Brussels, Belgium

**Keywords:** Cost-of-illness analysis, Overweight, Obesity, Public health

## Abstract

**Background:**

People with overweight or obesity are at increased risk for disease later in life which cause important health costs.

The aim of this study was to estimate the health status and the corresponding costs in a sample of females with overweight or obesity which were participating in a Randomized Controlled Trial (RCT) exploring the effect of lifestyle habits changes on ectopic adipose tissue.

**Methods:**

Sixty-two non-diabetic premenopausal females without major comorbidities of overweight and obesity were recruited among patients visiting endocrinologists at the obesity clinic of the University Hospital of Antwerp and the University of Antwerp.

A RCT-embedded cost-of-illness approach with societal perspective, based on self-reported questionnaires and cost diaries (3 months recall) was applied to estimate the prevalence of different comorbidities and the related direct and indirect costs in this sample of overweight or obese females. The European Quality-of-Life-5D questionnaire was used to define the health state and the corresponding utility index of the participants.

**Results:**

The average direct health costs and health utilities observed in this sample were comparable with the general Flemish female population. This may partially be explained by the strict inclusion criteria of the RCT (i.e. overweight or obesity without diabetes type 2 or cardiovascular diseases). However, 15% of the participants had five or more comorbidities resulting in higher average costs and lower average health utility as compared to the general population, only 3 participants were diagnozed with the metabolic syndrome. In this subsample productivity was low due to high average absenteeism, yielding important total costs for the society.

**Conclusion:**

Secondary prevention to avoid health deterioration in overweight or obese females without major comorbidies is needed to contain health care costs.

**Trial registration:**

ClinicalTrials.gov: NCT02831621, approval of the ethics committee of the University Hospital of Antwerp (number: 14/17/205 -ref: 7543075363).

## Background

The increasing prevalence of overweight and obesity during the last decades has become a serious public health concern and has placed a financial burden on the wider economy [1, 2].

The association between overweight or obesity and health disorders such as the metabolic syndrome has been well established.

In Flanders, the northern Dutch speaking part of Belgium, the prevalence of overweight (BMI ≥ 25 kg.m^− 2^) and obesity (BMI ≥ 30 kg.m^− 2^) in the female population (aged 35 to 44 years) is estimated at 42.5 and 11.4% respectively [3].

The extent to which overweight and obesity related metabolic abnormalities are seen defines the patient’s global cardiometabolic risk profile [4]. In this regard, a distinction is made between metabolically healthy obesity (MHO) and obese people suffering from the metabolic syndrome [5]. The prevalence of the metabolic syndrome in a population increases in people with higher BMI and age and is a marker for high health care utilization and costs in people with overweight [6, 7].

In general, overweight and obesity are associated with high direct and indirect costs. Direct costs include the costs of the diagnosis and treatment of overweight and obesity and especially their associated diseases and complications, while the indirect costs are those resulting from productivity loss such as work absenteeism, early retirement, and the lost value of life due to premature mortality [8, 9]. In European countries, this cost may range from 0.7 to 0.8% of the total annual expenses of the health insurers [10].

Although, overweight resulted in 7.4% of the Disability-Adjusted Life Year (DALY) in Belgium in 2004, the cost of overweight or obesity is as yet not well understood [10].

Especially information on the health costs in the overweight or obese population without major comorbidities is still lacking. The economic burden of a disease or condition can be estimated by cost-of-illness studies [11]. In general two approaches are used in cost-of-illness studies, namely the prevalence-based and the incidence-based approach. Prevalence-based studies estimate the costs associated with past and current consequences of the disease or condition in a given time period, typically a year. The incidence-based approach estimates the costs and consequences associated with new cases of the disease or condition in the current and future years [12]. Insights into the economic impact of overweight and obesity are important to strengthen the knowledge of the current burden associated with this condition and may inform decision makers to understand the scale of overweight or obesity related problems. This may help them to establish evidence-based public health policies to tackle the overweight and obesity problem.

The aim of this paper was to estimate the health status (i.e. the comorbidities) and the corresponding costs in a sample of 62 premenopausal non-diabetic overweight or obese females in Flanders. This paper was the first step towards a complete cost-effectiveness and cost-utility analysis of an intervention (diet and physical activity) to reduce body weight, abdominal and ectopic adipose tissue in this sample.

## Methods

### Theoretical framework

**Fig. 1:**
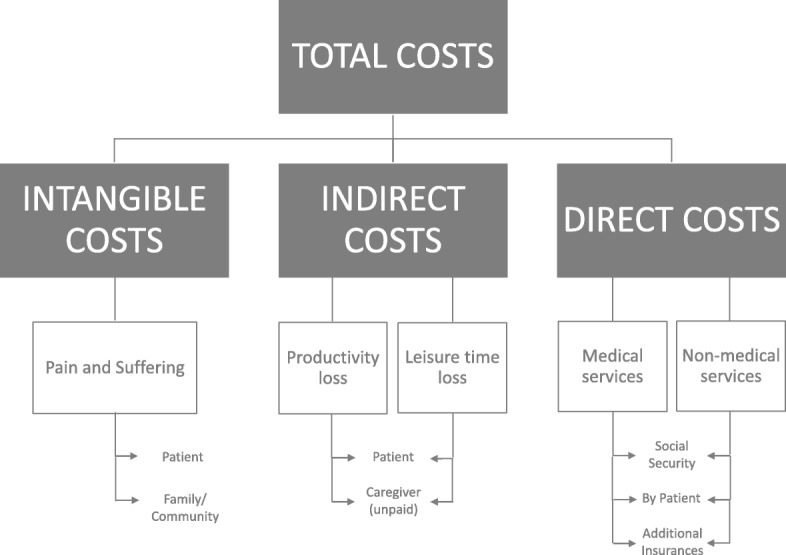
Components of cost analysis

Figure 1 depicts the theoretical framework that was followed in this study. A bottom-up cost-of-illness approach was applied to estimate the prevalence of different comorbidities and the related direct and indirect costs in this sample of overweight and obese females. Direct medical costs include expenses for visits to physicians or medication. Direct non-medical costs include for example the use of braces if needed in conditions of morbid obesity. Indirect costs encompass productivity loss (e.g. premature mortality, absenteeism or presenteeism) or leisure time loss. Intangible costs refer to costs related to pain or psychological suffering (e.g. stigmatization), however the latter is difficult to measure and will not be analysed in this present study.

A prevalence-based approach was used to examine the costs of overweight and obesity in premenopausal women in the Flanders region. A prevalence-based study estimates the economic burden of a disease or condition over a well-defined time period, in this case three months [13].

### Study participants

The study was conducted in the larger Antwerp metropolitan area in Flanders.

Study participants were recruited among patients visiting endocrinologists at the obesity clinic of the University Hospital of Antwerp between May 2016 and August 2016. In addition, poster recruitment was performed in the University Hospital of Antwerp and the University of Antwerp. A total of 120 participants responded and wanted to volunteer in this study.

Table 1 shows the inclusion and exclusion criteria for the selection of the study participants. All included participants were non-diabetic premenopausal overweight or obese females. Since fitness training was part of the intervention and an individually but standardized exercise scheme was followed, women were excluded when suffering from cardiovascular diseases or musculoskeletal disorders that made strength or endurance training impossible. This selection procedure resulted in a sample size of 62 participants.

**Table 1:** Inclusion and exclusion criteria of participants

Inclusion	Exclusion
women	planned pregnancy within one year
BMI > 27 kg/m^2^	hypothyroidism
age > 18 years	diabetes type 2 or prediabetes with medication use
stable body weight, i.e., not varying by > 3% for at least 3 months prior to the first consultation	changes in medication regimen which can affect study outcomes (e.g. of lipid-lowering or antihypertensive agents)
premenopausal state defined by hormonal data; FSH > 25 mU/ml and estradiol < 20 pg/ml	using drugs known to affect body weight and lipid distribution including tricyclic antidepressant agents
willing to participate in a lifestyle based weight loss intervention (diet or exercise)	abuses alcohol or has a history of alcohol abuse, i.e., more than 2 alcoholic consumptions/day or binge drinking
no physical dysfunctions which makes increased physical activity impossible	exclusion criteria related to MRI and CT
able to read and understand the guidelines given by the dietician and sign the informed consent	Serious problems or diseases limiting the performance of a standardized training protocol, for example serious osteoarthritis, heart ischemia, …

All included participants (*n* = 62) were randomized to a hypocaloric dietary intervention group or a combination group of hypocaloric diet and increased physical activity and they were measured at baseline, in between (3 months) and at the end of the intervention (6 months). After the intervention period, participants were followed up during 6 months and measured in between this period (9 months) and at the end of the study (12 months).

This study was the first step of a trial-embedded health economic evaluation. Its protocol has been described and published elsewhere [14]. The protocol and consent forms were approved by the ethical committee of the University Hospital Antwerp (approval number: 14/17/205). All participants gave prior written consent.

### Data collection

Data were gathered by means of self-reported questionnaires (cost diary) at baseline, during the intervention and follow up. In this paper, results from baseline questionnaires (3) are discussed. Questionnaires were based on similar questionnaires used in a study from the University of Ghent in which costs in an obese population in Flanders were reported [15]. In this way, our results could be compared with a reference group of obese people.

One questionnaire with closed questions aimed at assessing the type of comorbidities over the last three months. A set of overweight-related (e.g. hypertension, cardiovascular disorders, gall bladder; cancer; arthritis or osteoarthritis, depression) and pathologies unrelated to overweight (e.g. allergy, asthma, chronic obstructive pulmonary disease (COPD), skin disorders, hypo/hyperthyroidism, stomach ulcer, migraine headache) was listed. Respondents could answer “yes”, “no” or “previously”. One open question offered the opportunity to add “other disorders”. A final question asked if the physician gave advice to start a treatment for overweight or obesity. This could be answered by “yes” or “no” while mentioning a date.

Another questionnaire focused on the assessment of direct and indirect costs. Units consumed over the last three months as well as prices were asked for visits to health care providers such as physicians, specialists, nurses, dieticians and physiotherapists. Consumption units and prices of medication were also asked retrospectively over the same period. Furthermore, consumption and prices of hospitalization, day hospitalization, surgery and special diagnostics or medical examinations were assessed over the same period together with the number of days absent from work due to any health problems.

The third questionnaire was the European Quality-of-Life-5D (EQ-5D-5 L). The EQ-5D consists of five dimensions (mobility, self-care, usual activity, pain/discomfort, anxiety/depression) on a five level scale (ranging from 1 = no difficulties to 5 = extreme difficulties) allowing to define a health state and the calculation of a corresponding utility index (as a proxy of quality of life). A utility of 1 is equal to perfect health, while 0 stands for death [16]. Questionnaires were given to the volunteers during the first meeting in the laboratory. Participants were asked to fill out the questionnaires retrospectively (for the last three months) and to return the completed questionnaires when visiting the laboratory for a second time one week later.

### Data analysis

Data management was conducted in three steps. In a first step one researcher (JB) manually imputed the results from the questionnaires into a spreadsheet (Microsoft Excel 2013). In a second step two researchers (WH, JT) checked each cell value of the spreadsheet with the questionnaire and corrected the value if needed. In a third step, one researcher (JT) conducted a final round of ad random data checking.

Data from the comorbidity questionnaire were analysed using a frequency analyses.

Direct medical cost data were presented in 2017 Euros and all units consumed and unit prices were reported in a non-aggregated form. If participants did not mention unit prices of care providers, the fees determined on 01/01/2017 were searched on the website of the national institute for health and invalidity assurance (RIZIV) [17–19].

It was assumed that patients consulted accredited physicians. For assessing the costs of a specialist doctor, the billing service tariff of the University Hospital of Antwerp was applied. According to RIZIV, the federal institution that organises the mandatory health insurance, the price for a dietician consultation amounts to 19.66 Euro. In the present study, this is an out-of-pocket expense for the patient, because such a visit is reimbursed by Flemish health insurers only in case of diabetes. It was assumed that the participants visited an accredited physiotherapist for a 30 min treatment. This price per visit amounts to 22.26 Euro of which 16.37 Euro is reimbursed from the insurer (in case the therapist joined the RIZIV convention and the patient has right to the normal reimbursement rate), resulting in a cost for the patient of 5.89 Euro per visit [20]. Respondents mentioned no nursing costs, hence these were not valued.

Medication costs were calculated based on the units consumed through the Belgian commented online drug compendium [21]. Costs for hospitalization, surgery, day-care hospitalization and medical examinations or diagnostic testing were based on the billing service 2017 tariff of the University Hospital of Antwerp. A mean cost of one day in a hospital in the Antwerp region was set at 673.00 Euro (medical services not included). No direct non-medical costs were reported, hence these were not valued.

Absenteeism was analysed as an indirect cost and valued as 288.00 Euro per day following the whitepaper of SECUREX, a company providing social secretary services in Flanders [22].

The costs were reported over a three months time span. Costs were analysed from the perspective of the patient, the health insurer and the society respectively.

Finally, the EQ-5D questionnaire was analysed using the EQ-5D-5 L crosswalk index value calculator with social UK tariff [16].

Statistical analysis was performed using SPSS Version 22.0 (IBM SPSS Statistics V22.0). As could be expected, cost data in this study were not normally distributed. For pragmatic reasons however, results were presented as means and the corresponding 95% confidence intervals. To analyse the association between BMI, utility score, age, number of comorbidities and the cost, Spearman rank order correlation coefficients were calculated. Statistical significance was set at 5%.

## Results

A total of 59 out of the 62 females returned the questionnaires, which were analysed for this study. Their mean age was 37 years (ranging from 19 to 53 years) with a mean body mass index of 32.6 kg.m^− 2^ (measured values, range from 27 to 44 kg.m^− 2^). Most of the participantswere obese (86%) and were residents of the Antwerp metropolitan region (82%) in Flanders living outside of the city (62%). About 47% of the participants hold a higher education (no university) degree while 18% were university degree holders. In the total sample, four participants (7%) were unemployed at baseline but two of them found a job during the course of this intervention study. Also, four students were included in this study.

On average, a participant suffered from about three comorbidities. Only four responders were free of any comorbidity while six volunteers showed six to eight comorbidities. Besides diabetes, which was an exclusion criteria, the following comorbidities of the questionnaire were not reported: any form of cancer, COPD, liver cirrhosis, chronic hepatitis, Parkinson disease and HIV. Since only three subjects had the metabolic syndrome, the majority of our sample (95%) were metabolically healthy overweight or obese women [5]. Table 2 presents the relative prevalence of the reported comorbidities in this sample. Musculoskeletal disorders and allergies seemed to be the most prominent comorbidities, but also depression, hypertension, skin problems and migraine headache were highly prevalent. As « other disorders » were mentioned: reflux oesophagitis, hypercholesterolemia, lupus erythematosus, low limb lipoedemia, glaucoma, eczema (all disorders mentioned once). With the exception of thyroid disorders, all listed diseases showed higher prevalence in the sample under investigation as compared to a reference sample of 1547 representative for the adult Flemish female population [23]. In 59% of the cases it was the general practitioner who suggested to start a treatment against the patient’s overweight or obesity.

**Table 2:** Relative prevalence (%) of reported comorbidities in this sample in reference to the prevalence (%) in the general female population in Flanders [23]

Comorbidity	Study sample	Reference of flemish females [23]
	Prevalence (%)	Prevalence (%)
Hypertension	22.0	18.6
Cardiovascular problems	1.7	1.3
Gall bladder problems	6.8	1.0
Arthritis or osteoarthritis	16.9	10.6
Depression	39.0	7.6
Incontinence	8.5	6.2
Back and neck problems	62.7	25.2
Joint problems	40.7	21.1
Ovarian cysts	6.8	^a^
Allergy	47.5	16.6
Asthma	3.4	3.1
Skin	28.8	3.3
Thyroid problems	3.4	6.9
Hernia inguinal	10.2	^a^
Stomach ulcer	13.6	1.4
Kidney stones	5.1	0.5
Migraine	27.1	14.0
Epilepsy	1.7	0.9

^a^unknown

On average, respondents consulted a physician once over the last three months [95%CI: 0.7 to 1.3]. Average number of visits to a specialist doctor were also reported to be once in three months [95%CI: 0.4 to 1.6]. In this three-month period before the start of the intervention, only three respondents went to a dietician. Of those, two participants went twice while one person went once. No visits to a nurse were reported. During this three-month period the average number of visits to a physiotherapist was two [95%CI: 0.8 to 3.5].

Medical examination and diagnostic testing over the last three months was reported by 29% of the respondents. A second examination or test was consumed by 5% of the participants. Hospitalization was needed for 7% of the study participants. For those, length of stay in the hospital was one (*n* = 3) to two (*n* = 1) nights. Two patients needed surgery, both for shoulder problems. Three volunteers reported a visit to a day-care hospital for sleep disorder, liver biopsy and shoulder problems.

About 65% of the participants were on medication during the past three months. When contraceptives were not taken into account the prevalence of drug consumption was reduced to 55%. Two different drugs were consumed by 42% of the respondents while 5% of the volunteers consumed seven different drugs. Table 3 shows the indications and the relative prevalence of drug consumption in the sample. Pain reducing drugs were consumed by 53% of the sample while medication against depression and hypertension ranked on the second place (both 17%).

**Table 3:** Indications and the relative prevalence (%) of drug consumption in this sample in reference to the general female prevalence (%)in Flanders [23]

Indication	Study sample	Population [23]
	Prevalence (%)	Prevalence (%)
Pain	53.3	3.5
Depression	16.7	3.1
Cardiovascular problems	16.7	11.9
Allergy	13.3	2.4
Stomach problems	11.7	2.7
Infections	6.7	1.6
Endocrine problems	3.3	2.7
Dietary supplements	3.3	^a^
Birth control	10.0	4.2
Inflammation	13.3	3.6

^a^unknown

Absenteeism was reported by 13.6% of the participants. The average number of days absent from work in this sample over the past three months was 5.5 days [95% CI: 0.3 to 10.7]. Three participants reported 90 days absenteeism for reasons of depression while one person remained absent from work during 30 days because of low back pain.

Table 4 shows the average direct and indirect costs over the past three months from the perspective of the patient, the insurers and the society. On average, a participant of this sample has spent 62.60 Euro for health care while health insurers paid 280.20 Euro over the past three months period. If absenteeism is taken into consideration, the total societal cost for this sample of premenopausal non-diabetic females over the last three months was 2239.7 Euro.

**Table 4:** Average direct and indirect costs (€) over 3 months from the perspective of the patient, the insurers and the society measured by recall (3 months) cost diaries

Costs	Units consumed	Price patient	Price insurer	Price society
Direct costs				
Physician	1.0	4.1	21.4	25.5
Specialist doctor	1.0	11.8	38.9	50.7
Dietician	0.1	2.7	0.0	2.7
Physiotherapist	2.1	12.5	34.7	47.2
Medication	^a^	9.7	14.3	23.9
Hospitalization and surgery	0.1	14.5	52.9	68.4
Day-care hospitalization	0.05	3.0	66.0	69.0
Medical examination	0.3	4.3	52.0	56.3
Subtotal direct costs		62.6	280.2	343.7
Indirect costs				
Absenteeism	5.5	0.0	0.0	1552.3
Total direct + Indirect costs		62.6	280.2	2239.7

^a^ unknown, all costs are expressed in Euro (€)

The calculated average utility index was 0.83 [95% CI: 0.79 to 0.87]. About 27% of the participants reported to be in « full health » (i.e. utility index = 1) while 13.5% had a utility index score lower than 0.70. One person reported a utility index of 0.34.

Table 5 depicts the Spearman rank order correlation coefficients matrix between costs, utility index, BMI, age and number of comorbidities. The results suggested that societal cost in the sample is negatively but significantly related to utility scores (i.e. self-reported health status) while a positive association (albeit not statistically significant) between costs for the society and the number of self reported comorbidities, age and body mass index were found. Utility was negatively and significantly associated with comorbidity, cost of absenteeism as well as costs from the different perspectives and BMI, a negative association (*p* > 0.05) was observed with age. The number of comorbidities was positively related with cost for the patient (*p* < 0.05) and cost for the insurer and society (*p* > 0.05) as well as cost from absenteeism (*p* > 0.05). The correlation between number of comorbidities and BMI was positive and statistically significant.

**Table 5:** Spearman rank order correlation coefficients (*Rho*) matrix between costs, utility index, BMI, age and number of comorbidities in this study sample

	Utility	Cost absenteeism	Total cost patient	Total cost insurer	Total cost society	BMI	Age	Number comorbidities
Utility	1.000	−0.395**	−0.532**	−0.501**	−0.550**	−0.303*	−0.249	−0.443**
Cost absenteeism	−0.395**	1.000	0.359**	0.371**	0.585**	0.140	0.178	0.201
Total cost patient	−0.532**	0.359**	1.000	0.895**	0.886**	0.197	.291*	0.260*
Total cost insurer	−0.501**	0.371**	0.895**	1.000	0.929**	0.222	0.194	0.201
Total cost society	−0.550**	0.585**	0.886**	0.929**	1.000	0.182	0.189	0.190
BMI	−0.303*	0.140	0.197	0.222	0.182	1.000	0.017	0.394**
Age	−0.249	0.178	0.291*	0.194	0.189	0.017	1.000	0.168
Number comorbidies	−0.443**	0.201	0.260*	0.201	0.190	0.394**	0.168	1.000

*: correlation is significant at the 0.05 level; **: correlation is significant at the 0.01 level

## Discussion

Overweight and obesity are important public health issues and may impose a significant health economic burden to a society. In this study health status and related costs of 59 premenopausal non-diabetic overweight or obese (BMI > 27 kg.m-2) females in Flanders were assessed using a three months self-reported recall questionnaire. All results are compared with reference data of the general female population in Flanders [3, 22, 23] or reference data from a COI in obese men and women in the larger Ghent area [15].

Although the majority of the sample (95%) encompasses the so-called MHO, the average number of the self-reported comorbidities was striking. Compared to the general female population in Flanders, prevalence of all listed disorders was a factor 0.1 to 10 higher in the sample under investigation except for thyroid disorders which was an exclusion criteria. (Table 1) [23]. The most prevalent complaints were back and neck disorders (62.7%) and joint problems (40.7%). The findings in this study corroborate the results of a U.S. study in which 312 obese adults reported to suffer mainly from low back pain (50%) and joint pain (28%) [24]. The fact that depressed mood status was about 5 times more prevalent (39%) in the sample under investigation than in the general adult female population (7.6%) [23], supports the results of a national survey of obese persons in Sweden. This survey concluded much higher levels of anxiety and depression in overweight and obese, as well as poorer perceived health compared with a healthy, non-obese reference group [25]. Thus, such sample of overweight and obese females shows higher comorbidity prevalence compared to the general female population, which may incur higher health costs [7].

Although diabetes type 2 and cardiovascular diseases were excluded, the prevalence of other overweight and obesity related comorbidities in this sample was associated with increased drug consumption (Table 3). Our findings suggest an even more important medication use in people with overweight and obesity than was reported by Raebel et al. who concluded that on average a person with obesity compared to a non-obese person consumes 1.81 times more prescription drugs over a one-year time period [26]. Yearly costs for medication in the sample was about 96.00 Euro.

The costs from institutionalized care such as hospitalization and diagnostic examinations totalled on average 774.80 Euro in this study which are higher than those in the healthy reference population (571.00 Euro) [23]. From a societal perspective, the yearly costs for visiting health care professionals in this study was 504.40 Euro. This amount is similar as compared to the same cost in the general Flemish population (527.00 Euro), but the contribution of consultations to a specialist doctor was higher in our sample in comparison with the general female population in Flanders [23].

The average yearly direct medical costs from the societal perspective totalled 1374.80 Euro in the present sample of non-diabetic women with overweight or obesity which is only about 30% of the total direct costs reported in the Ghent study [15]. In the latter study, performed in 62 obese diabetic and non-diabetic patients, similar cost diaries were used. In the Ghent study, yearly costs visiting health care providers (822.00 Euro), yearly medication costs (300.00 Euro) and yearly costs of institutionalized care (3200.00 Euro) were much higher than in our study sample. [15]Main reason for the aberrant differences between both studies may be the strict inclusion and exclusion criteria in our study that prevented people with serious health problems (e.g. ischaemic heart disease and diabetes) to participate in this study. It has been described that ischaemic heart disease and diabetes are the main contributors to the high costs of obesity, probably because ischaemic heart disease treatment is expensive and diabetes is a disease that has the highest incidence risk in people with obesity [27, 28]. Besides this, the mean BMI and age in the Ghent study was higher and might have lead to higher costs [29].

Based on extrapolation of data of three months, participants of this study were on average 22 days absent from work per year. This absenteeism rate is much higher than that in the general population (14 days) [22]. The simple extrapolation from three months of data towards twelve months may have led to a bias of the yearly absenteeism. However, this finding is consistent with the findings of Seidell et al. who found increased absenteeism in obese females because of medical problems such as mental health disorders or musculoskeletal problems [30]. Both types of disorders were also prominent in the sample of the present study. As the correlation matrix showed (Table 5), productivity-loss may be an important cost driver for the society and resulted in this sample in a yearly average indirect cost of 6208.00 Euro.

Participants in the present study showed a mean utility index score of 0.83 (SD = 0.141). This value is similar to the utility score of the general population in Flanders [3] and somewhat higher than the pilot study of the larger Ghent area which showed a mean utility index score of 0.82 (SD = 0.12) [15]. About 13.5% of the sample showed low utility index scores (< 0.70). Such scores are comparable with utility index scores reported by patients with Parkinson disease (utility index = 0.58) [31] or severe rheumatic disorder (utility index = 0.57) [32].

The sample in the present study consisted predominantly of the so-called MHO [33] which resulted in average utilities and direct costs that were very similar to the general population but importantly different from those of the obese reference sample [15]. This can be illustrated by the fact that people were excluded when they had musculoskeletal problems that made strength or cardiovascular training impossible leading to exclusion of severe osteoartitis. Also, it is stated that an increased BMI is associated with reduced health-related quality of life, even in the absence of metabolic comorbidities [34].

Four out of 59 participants reported high productivity losses that resulted in yearly indirect costs of more than 103,680.00 Euro per person.

Nine out of the 59 participants showed five or more comorbidities. In this subsample average costs increased to 360.00 Euro (patient’s perspective), to 1256.00 Euro (insurer’s perspective) and 10,160.00 Euro (societal perspective) while average utility was decreased to 0.73 (ranging from 0.45 to 1.00). In this regard, it can be confirmed that MHO do have an increased risk of diabetes type 2 and coronary diseases in later life which indicates that healthy obesity can be described as an intermediate stage of disease progression accompanied with important health costs in later life [35, 36].

In this sample, a higher rate of unemployment was seen in comparison with general female unemployment rates in Flanders (7% vs. 4.5%). Although it is assumed that there is a negative effect of obesity on employment, mediated through disability resulting from the accumulation of chronic conditions, there is also a possibility that pre-existing conditions contribute to obesity at baseline [37, 38].

Health-related costs in overweight or obese residents in Flanders are as yet no well documented. This study adds information to the findings of a pilot study that was previously conducted using similar methods in a sample of 62 obese males and females of the Ghent area in Flanders [15]. Combined data and results of both studies may help Flemish policy makers during their decision making processes when planning actions to contain the burden of overweight and obesity. For example, the findings of this study may be used by health economists as input data when modelling is needed in cost-effectiveness studies. According to a recently conducted research in Europe, overweight is responsible for 20–26% of the direct medical costs [28]. Research in various countries showed that 2–5% of annual healthcare costs are attributable to overweight [8, 39–43].

This study was conducted on a sample that was recruited to participate in a prospective randomized controlled trial. Hence it was a captive sample, which may explain the high response rate (59 out of 62) and helped the researchers to ask participants if they had doubts about the reporting or in case of missing values. Despite this advantage, underreporting or overreporting can never be excluded although it is reported in literature thatthe reconstruction of total costs based on self-reported costs (cost diary) shows good agreement with data from other sources such as from insurance companies [44, 45].

Another limitation of this study is the fact that intangible costs were omitted. For example the use of pain medication was considerable in this sample, but the subjectively felt “pain and suffering” was not calculated. Similarly, tangible and intangible costs due to discrimination, bullying or stigmatization were not calculated [46]. Since welfare losses were not valued, the total economic burden of overweight and obesity may be underestimated [47]. It is recommended that future studies examining the economic impact of overweight or obesity also examine the intangible costs to catch the full economic burden [48]. It can be suggested to determine non-financial welfare costs using disability adjusted life years (DALYs) [49, 50].

In the sample under investigation, the average yearly total cost from a societal perspective was 8958.8 Euro. The fact that data from three months were extrapolated to a whole year, might have led to a bias. Since no appropriate recall periods are determined in cost-of-illness studies, it is hard to identify whether it is better to collect a short-term snapshot of resource use and extrapolate to a longer period or to ask questions covering a broader period which potentially increases recall bias or leads to more uncertainty and hance higher variability of results [51, 52]. Often, costs are calculated based on the reported rates of comorbidities, or data from an insurance company [40, 53]. In this study, a method was used in which all costs were reported by participants over a short period of three months. Extrapolation of the short-term data (three month) to one year was used because of its patient-friendliness and proven validity [44].

Finally, this sample consisted MHO females without diabetes or any other major comorbidity. Thus, the cost results in this study are likely to underestimate the true costs on population level of adult women with overweight and obesity.

## Conclusion

On average, “healthy overweight or obese” non-diabetic premenopausal females (mean age 37 years) of the Antwerp metropolitan area showed similar direct health costs and health utility as compared to the general Flemish female population. The observed high number of comorbidities and drug intake did not primarily lead to an increased average direct medical cost. In contrast, average absenteeism was high in this sample and resulted in important total costs from the societal perspective.

Overweight or obese persons are at increased risk for disease such as diabetes mellitus type 2 later in life, which may then cause even more important health costs. A subsample of nine participants with five or more comorbidities showed already high costs and low health utility as compared to the general population. Thus, secondary prevention to avoid health deterioration in MHO females is needed to contain health care and social costs.

## Abbreviations

BMI: Body mass Index (kg/m^2^)
DALY: Disability-adjusted life year
MHO: Metabolically Healthy Obesity
RCT: Randomized controlled trial
RIZIV: Rijksinstituut voor ziekte- en invaliditeitsverzekering
